# Inconsistencies in the specific nucleobase pairing motif prone to photodimerization in a MOF nanoreactor

**DOI:** 10.1038/s41467-021-27196-6

**Published:** 2021-11-30

**Authors:** Pascale Clivio

**Affiliations:** Université de Reims Champagne Ardenne, Institut de Chimie Moléculaire de Reims, CNRS UMR 7312, UFR de Pharmacie, 51 rue Cognacq-Jay, F-51096 Reims Cedex, France

**Keywords:** Nanopores, Metal-organic frameworks

**arising from** S. L. Anderson et al. *Nature Communications* 10.1038/s41467-019-09486-2 (2019)

Recently, Anderson et al.^1^ reported the synthesis of an adenine-tagged metal-organic framework (MOF), namely SION-19, and proposed a binding mode of its adenine residues to thymines based on computational studies and supported through the experimental determination of the thymine-dimerized UV photoproduct stereomer. The thymine adenine binding mode calculated by the authors affords the *trans-anti* photoproduct. This base-pairing mode is in contradiction with their experimental results since their dithymine photoproduct is not the *trans-anti* but the *cis-syn* isomer. For SION-19 to be used in any supramolecular recognition of thymine, a thorough study is necessary.

**Fig. 1:**
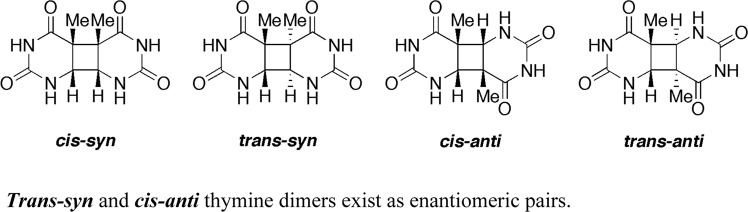
Structure of the four diastereomers of the cyclobutane thymine dimer. ***Cis*** (endo) and ***trans*** (exo) stereomers; ***syn*** (head-to-head) and ***anti*** (head-to-tail) regioisomers ***Trans***-***syn*** and ***cis***-***anti*** thymine dimers exist as enantiomeric pairs.

The MOF reported by Anderson et al.^1^ and aimed at supramolecular recognition of thymine in view of biomimetic applications is composed of Zn^II^(adeninate) columns mutually interconnected via coordination to TBAPy (1,3,6,8-tetra(4-carboxylphenyl) pyrene) ligands. It displays surface cavities lined with adenine residues presenting their Watson-Crick face. In this publication, thymine is reported to diffuse through the pores of SION-19 and to be specifically self-oriented, at 40–45% loading, by base pairing with adenine residues so as to be packed in a geometrical arrangement suitable for photodimerization. To identify this geometrical arrangement, the authors have studied, using a computational approach (Density Functional Theory and Molecular Dynamics simulation), the base-pairing pattern between the thymine and adenine residues at the pore surface of SION-19. According to their calculation, thymines bind to adenine residues through a reverse Watson–Crick base pairing mode (see Fig. 3c in ref. ^1^) and are mutually arranged in a respective head-to-tail (i.e. *anti*) geometric orientation (see Fig. 3c, d in ref. ^1^) with a *trans* facial diastereoselectivity (see Fig. 3c in ref. ^1^). Such thymine organization would consequently afford, upon UV exposure, a photodimer whose stereochemistry is presented by the authors as being *trans-anti* (see Fig. 4c in ref. ^1^ and Fig. 1).

What is disturbing in the report of Anderson et al.^1^ is that the authors provide evidence (see Fig. 3c, d and 4 in ref. ^1^) clearly indicating that the photodimer is *trans-anti*, when, contradictorily, they experimentally establish the structure of the pore-derived thymine dimer not to be the *trans-anti* stereoisomer but a head-to-head *syn* regioisomer (see Fig. 5 in ref. ^1^, Fig. 1), meaning that the computational work presented to support the nature of the base pairing, the respective orientation of the thymines, and the resulting thymine photodimer is irrelevant and in contradiction with the experimental results.

Indeed, in their experimental work, the authors have prepared, as an authentic sample, the thymine dimer obtained from 254 nm UV irradiation of a frozen thymine solution, unambiguously assigned as the *cis-syn* isomer (Fig. 1) by Blackburn and Davies^2^ in 1965. The authors have observed that the thymine dimer formed in the MOF pore and the authentic *cis-syn* isomer have identical retention times in UHPLC and identical parent and fragmentation ion peaks on electrospray ionization mass spectra. Importantly, a fragment observed at *m/z* 210 on the UHPLC-ESI/MS spectra of the SION-19-derived photoproduct (see Supplementary Figs. 36, 37 and 41 in ref. ^1^) is characteristic of a *syn* thymine dimer isomer (Fig. 1)^3^. This would indicate that the pore-derived thymine dimer is indeed a *syn* isomer (*cis-syn* or *trans-syn*, Fig. 1), in contradiction with the computational model of adenine thymine pairing in SION-19, allegedly presented as affording the *trans-anti* thymine dimer.

In other words, the theoretical work presented by Anderson et al.^1^ is not relevant to elucidate the nucleobase pairing in SION-19.

The bias in this study likely results from the exclusive computation of a reversed Watson–Crick base-pairing mode for the thymine adenine binding configuration (see Fig. 3c and supplementary Fig. 17 in ref. ^1^) even though the authors claim the presence of a Watson–Crick interaction: “*…*stabilisation of Thy via W-C H-bonding with Ade allows it to conform to both the Smith and Woodward rules.” If indeed the reversed Watson–Crick mode of pairing has been shown to be favoured in an adenine-based MOF^4^, the authors’ interpretation is called into question by their failure to consider the canonical Watson–Crick base-pairing mode as well as an alternative binding mode^5^ in their computational analysis.

In addition to computing all binding modes of thymine with the Watson–Crick face of the adenine residues, to unambiguously determine the structure of the thymine dimer produced in the pore of SION-19 under UV exposure, the four thymine dimer stereoisomers (*cis-syn*, *trans-syn*, *cis-anti* and *trans-anti*, Fig. 1), which fortunately have already been shown to elute differently in HPLC^6–8^, should be prepared by acetone photosensitization in aqueous solution^6–8^, then individually compared by UHPLC/ESI/MS with the MOF-derived photodimer.

In conclusion, as SION-19 is claimed to be a bio-nanoreactor due to its “*…* ability to ‘lock’ molecules in specific positions that can be subsequently dimerized upon light irradiation*…*”, it is crucial to rigorously determine the specific mode of binding between the host and guest at the supramolecular level and the stereochemistry of the dimerized photoproduct. Currently, the work of Anderson et al. does not fulfil this task.
